# Draft Genome Sequence of the 1,4-Dioxane-Degrading Bacterium Pseudonocardia dioxanivorans BERK-1

**DOI:** 10.1128/genomeA.00207-18

**Published:** 2018-04-05

**Authors:** Angel A. Ramos-Garcia, Vijay Shankar, Christopher A. Saski, Tom Hsiang, David L. Freedman

**Affiliations:** a Department of Environmental Engineering and Earth Sciences, Clemson University, Clemson, South Carolina, USA; b Department of Genetics and Biochemistry, Clemson University, Clemson, South Carolina, USA; c Environmental Sciences, University of Guelph, Guelph, Ontario, Canada

## Abstract

Pseudonocardia dioxanivorans strain BERK-1 grows aerobically with 1,4-dioxane as its sole substrate. Reported here is its draft genome sequence, with a size of 7.1 Mbp. Key genes are highlighted in this article. BERK-1 exhibits a reduced level of cell aggregation and adherence to surfaces compared to those of P. dioxanivorans CB1190, giving it an apparent advantage for movement through soil.

## GENOME ANNOUNCEMENT

Pseudonocardia dioxanivorans BERK-1 was isolated from sediment and groundwater samples from an aquifer in South Carolina that is contaminated with 1,4-dioxane. BERK-1 is able to grow on 1,4-dioxane at concentrations of as high as 1,000 mg/liter, comparable to other microbes that use this contaminant as the sole substrate. Colonies of BERK-1 were grown on Bacto agar plates using ammonium mineral salts medium (AMSM) (1) amended with 1,4-dioxane and incubated at 30°C. Cells were sent to the Microbiome Core Facility (https://www.med.unc.edu/microbiome/) at the University of North Carolina at Chapel Hill, where DNA was extracted from a single colony.

The genomic DNA of BERK-1 was fragmented and prepared into a sequence library using a DNA library preparation kit with barcoding (Illumina, San Diego, CA, USA). This library was sequenced by 150-bp paired-end sequencing using the Illumina Sequencing MiSeq PE150(300) system, which produced 4,179,425 reads with a yield of 627 Mb. The sequencing output from the Illumina MiSeq platform was converted to fastq format and demultiplexed using Illumina bcl2fastq version 2.18.0.12. Illumina adapters were trimmed, and reads were quality filtered using Trim Galore (2). High-quality adapter-trimmed reads were *de novo* assembled using SPAdes 3.11.0 (3). The assembled genome was polished through Mauve (4) by using the genome sequence of P. dioxanivorans strain CB1190 as a reference. The polished genome was scanned for open reading frames (ORFs) in all 6 possible frames using Glimmer 3.02 (5). Identified ORFs were annotated using the latest SEED hierarchical database. The draft genome sequence for strain BERK-1 contains 219 contigs, accounting for a total of 7,073,226 bp (73.4% G+C content), with an *N*_50_ value of 61,756 bp and a maximum contig size of 268,190 bp. According to the Rapid Annotation using Subsystems Technology pipeline, BERK-1 contains 6,686 coding sequences (CDSs), genes across 425 subsystems, 44 tRNA genes, and 2 rRNA genes.

Similar to other 1,4-dioxane-degrading microbes, the BERK-1 genome includes tetrahydrofuran monooxygenase genes, which are likely responsible for the initial oxidation of 1,4-dioxane. Aldehyde dehydrogenase genes were also found; these are involved in the oxidation of aldehyde group intermediates in the proposed 1,4-dioxane degradation pathway (6). The number of genes associated with virulence factors and pathogenicity in strain BERK-1 is similar to what is present in strain CB1190 (7) and strain PH-06 (8–11).

### Accession number(s).

The draft genome sequence and annotation have been deposited in the DDBJ/ENA/GenBank database under the accession no. PJPW00000000. The version described in this paper is PJPW02000000.
